# Haplotype analysis of TP53 polymorphisms, Arg72Pro and Ins16, in BRCA1 and BRCA2 mutation carriers of French Canadian descent

**DOI:** 10.1186/1471-2407-8-96

**Published:** 2008-04-10

**Authors:** Luca Cavallone, Suzanna L Arcand, Christine Maugard, Parviz Ghadirian, Anne-Marie Mes-Masson, Diane Provencher, Patricia N Tonin

**Affiliations:** 1 Department of Human Genetics, McGill University, Montreal, Canada; 2 The Research Institute of the McGill University Health Centre, Montreal, Canada; 3 Service de Médecine Génique, Centre Hospitalier de l'Université de Montréal (CHUM), Montreal, Canada; 4 Département de médecine, Université de Montréal, Montreal, Canada; 5 Epidemiology Research Unit, Research Centre, CHUM – Hôtel-Dieu, Montreal, Canada; 6 Centre de Recherche du Centre Hospitalier de l'Université de Montréal/Institut du Cancer de Montréal, Hôpital Notre-Dame, Montreal, Canada; 7 Département d'obstétrique gynécologie, Division de gynécologie oncologique, Université de Montréal, Montreal; 8 Department of Medicine, McGill University, Montreal, Canada

## Abstract

**Background:**

The TP53 polymorphisms Arg72Pro (Ex4+199 G>C) and Ins16 (IVS3+24 ins16) have been proposed to modify risk of breast cancer associated with germline BRCA1 and BRCA2 mutations. Allele frequencies of these polymorphisms were investigated to determine if they modify risk in BRCA mutation carriers in breast cancer cases drawn from French Canadian cancer families, a population shown to exhibit strong founder effects.

**Methods:**

The frequencies of the TP53 alleles, genotypes and haplotypes of 157 index breast cancer cases comprised of 42 BRCA1 mutation carriers, 57 BRCA2 mutation carriers, and 58 BRCA mutation-negative cases, where each case was drawn from independently ascertained families were compared. The effect of TP53 variants on the age of diagnosis was also investigated for these groups. The TP53 polymorphisms were also investigated in 112 women of French Canadian descent with no personal history of cancer.

**Results:**

The BRCA mutation-positive groups had the highest frequency of homozygous carriers of the 72Pro allele compared with mutation-negative group. The TP53 polymorphisms exhibited linkage disequilibrium (p < 0.001), where the 72Arg and Ins16minus alleles occurred in strong disequilibrium. The highest frequency of carriers of Ins16minus-72Arg haplotype occurred in the BRCA mutation-negative groups. The BRCA1 mutation carriers homozygous for the 72Pro allele had the youngest ages of diagnosis of breast cancer. However none of these observations were statistically significant. In contrast, the BRCA2 mutation carriers homozygous for the 72Pro allele had a significantly older age of diagnosis of breast cancer (p = 0.018). Moreover, in this group, the mean age of diagnosis of breast cancer in carriers of the Ins16minus-72Arg haplotype was significantly younger than that of the individuals who did not this carry this haplotype (p = 0.009).

**Conclusion:**

We observed no significant association of breast cancer risk with TP53 genetic variants based on BRCA1/2 mutation carrier status. Although the small sample size did not permit analysis of all possible haplotypes, we observed that BRCA2 mutation carriers harboring the Ins16minus-72Arg haplotype had a significantly younger mean age of diagnosis of breast cancer. These observations suggest that investigations in a larger French Canadian sample are warranted to further elucidate the effects of TP53 variants on age of diagnosis of breast cancer among BRCA1 and BRCA2 mutation carriers.

## Background

Approximately 40% of French Canadian breast and/or ovarian cancer families have been shown to harbor germline mutations in the BRCA1 and BRCA2 cancer susceptibility genes [1-3]. At least five specific mutations in these genes have been found to recur in cancer families of French Canadian descent [2-5] and this has been attributed to common founders [1,3,6-9]. Germline mutations in BRCA1 and BRCA2 confer a high lifetime risk for breast and/or ovarian cancer, and early studies of familial cancer cases suggested that these risks may be as high as 80% [10,11]. However, lower estimates of lifetime risk for breast cancer of 66% in BRCA1 carriers and 45% in BRCA2 carriers were reported in subsequent population-based studies of pooled data [12,13]. Although various host factors may influence or modify risk, such as parity [14], genetic factors have also been proposed as modifiers of risk, such as genetic variants of HRAS1 [15], the androgen receptor (AR) [16], the 5'UTR of RAD51 [17], and repeat length polymorphisms in AIB1 [18], not all of which have been replicated or substantiated in subsequent studies [19-21].

Genetic variants of TP53 have received attention as possible modifiers of cancer risk due to the critical role of p53 in cell cycle control, DNA repair, and apoptosis, and possible interaction with BRCA1 and BRCA2 [22-24]. Germline mutations in TP53 also confer significantly increased risk for hereditary breast cancer in the context of the Li Fraumeni syndrome and Li Fraumeni-like syndrome families, however the overall contribution is less than that observed for BRCA1 and BRCA2, as was also shown in a recent study of French Canadian breast and/or ovarian cancer families [25]. The Arg72Pro (Ex4+199 G>C) and Ins16 (IVS3+24 ins16) TP53 polymorphisms have been extensively studied as putative breast cancer susceptibility variants with inconsistent results [26-42]. These variants have been shown to affect the *in vitro *apoptotic activity of p53 [43-45]. For example, the chemotherapeutic response was less favorable in ovarian cancer cases retaining the TP53 72Pro variant which possibly accounts for the overall poorer prognosis following treatment of such cases [46]. A significantly increased familial breast cancer risk for carriers of the Ins16 variant has also been reported [42].

Evidence is also emerging that the 72Pro and Ins16 TP53 polymorphisms may modify risk in carriers of BRCA1 or BRCA2 mutations [45,47]. The 72Pro allele has been found associated with a younger age of diagnosis of breast cancer in BRCA1 mutation carriers [47]. In a recent study of Spanish breast and/or ovarian cancer families, the haplotype lacking the Ins16 allele (referred to as "Ins16minus") and containing the 72Pro allele was associated with a younger age of diagnosis of breast cancer in BRCA2 mutation carriers than other comparative groups based on BRCA mutation status [45]. Recently we reported the frequency of TP53 polymorphisms, including 72Pro and Ins16 alleles in a study of the contribution of germline TP53 mutations in French Canadian breast and/or ovarian cancer families tested negative for BRCA1 and BRCA2 mutations [25]. However, the frequency of these polymorphisms and their effect on breast cancer risk in this founder population was not determined. Therefore, the aim of the present study was to investigate the contribution of these polymorphisms in a selected series of breast cancer cases with known BRCA1 and BRCA2 mutation status that were each drawn from independently ascertained breast and breast/ovarian cancer families of French Canadian descent. We report the frequencies of these alleles in BRCA1 and BRCA2 mutation-positive cases, and compare these frequencies with mutation-negative familial breast cancer cases, as well as female French Canadian controls with no personal history of cancer. The haplotype frequencies are also reported. In addition, we investigate the influence of TP53 alleles on the age of diagnosis of breast cancer.

## Methods

### Breast cancer cases and controls

Genotype analyses were performed on 157 breast cancer cases where each case was drawn from an independently ascertained cancer family in order to reduce bias due to familial relationships. Each family had at least three confirmed cases of female breast cancer (diagnosed ≤ 65 years of age), epithelial ovarian cancer, or male breast cancer as described previously [1-3,25]. The affected individuals in each family were first-, second- or third-degree relatives (occurring within the same lineage) to the index case that was selected for TP53 genotype analysis. Index cases reported grandparental French Canadian ancestry from Quebec, Canada. The index cases from 42 families were BRCA1 mutation-positive (age range at initial diagnosis of 27 to 65 years; 43.7 years mean age of diagnosis), from 57 families were BRCA2 mutation-positive (age range at initial diagnosis of 26 to 65 years; 42.7 years mean age of diagnosis), and 58 cases were mutation-negative (age range at initial diagnosis of 30 to 65 years; 47.2 years mean age of diagnosis) based on commercial sequencing service (Myriad Genetics^®^, Salt Lake City, UT). Within this group of index breast cancer cases there were nine breast cancer cases with primary cancers of the breast and ovary, of which six individuals were BRCA1 mutation-positive, two individuals were BRCA2 mutation-positive, and one case was mutation-negative. The families were ascertained through the Service de Médecine Génique, Centre Hospitalier de l'Université de Montréal (CHUM) and the Hereditary Cancer Clinics of McGill University in Montreal. The controls were comprised of 112 females participants with no personal history of breast or ovarian cancer ascertained from the French Canadian population of Quebec.

The clinical samples (pheripheral blood lymphocytes), and personal and family history were attained from the study participants at the Centre de recherche du Centre hospitalier de l'Université de Montréal – Hôpital Hotel-Dieu and Institut du cancer de Montréal with signed informed consent as part of the tissue and clinical banking activities of the Banque de tissus et de données of the Réseau de recherche sur le cancer of the Fonds de la Recherche en Santé du Québec (FRSQ). The study was granted ethical approval from the Research Ethics Boards of the participating research institutes.

### Genotype and haplotype analyses

The Arg72Pro polymorphism refers to the Ex4+199 G>C variant rs1042522 at nucleotide position 12139, which results in the nonsynonymous amino acid substitution of an arginine (72Arg) amino acid at codon 72 with a proline (72Pro) amino acid. The Ins16 polymorphism refers to the IVS3+24insACCTGGAGGGCTGGGG, the intronic variant rs17878362 at nucleotide position 11951. For simplicity genotypes lacking the Ins16 allele are referred to as "Ins16minus". The nucleotide position is based on the TP53 reference sequence X54156. The genotyping assays were performed on DNA extracted from peripheral blood leukoctyes. The Arg72Pro and Ins16 polymorphisms were assayed as described previously [25]. Haplotypes were determined as described in Osorio A. et al., 2006 [45].

### Statistical analyses

Allele, genotype and haplotype frequency distributions were compared by Monte-Carlo χ^2 ^analyses (Statistical Product and Service Solution Package, SPSS, Chicago, IL). Hardy-Weinberg equilibrium (HWE) for each polymorphism was tested by Monte-Carlo Markov Chain [48]. Linkage disequilibrium analysis and D' estimation were performed using the Arlequin v2.0 software package. A one-way ANOVA test was used to compare age related effects (onset of breast cancer). Kruskal-Wallis test was used where appropriate.

## Results

### Allele and Genotype frequencies in cases and controls

The genotype and allele frequencies of the TP53 polymorphisms from 112 controls and 157 index breast cancer cases each selected from an independently ascertained cancer family was determined. The controls had more individuals that were homozygous for the 72Pro allele than that of the cases, and this was also reflected in a higher 72Pro allele frequency (Table 1). In contrast, the cases had more individuals that were homozygous for the Ins16 allele than that the controls (Table 2). These findings are also reflected in the allele frequencies for these polymorphisms. However, the differences in genotype frequencies are not significant (Tables 1 and 2).

**Table 1:** Frequency of Ex4+199 G>C (72Pro) allele

		**Genotypic frequencies (%)**			***P *values**			**Allele frequencies (%)**		**HWE**
			
**Group**	**Sampl number e**	**GG (Arg, Arg)**	**GC (Arg, Pro)**	**CC (Pro, Pro)**	**All cases**	**BRCA2+**	**BRCA-**	**G**	**C**	***P *value**
**Controls**	112	57 (50.9)	46 (41.1)	9 (8.0)	0.84			160 (71.4)	64 (28.6)	1
**All cases**	157	80 (51.0)	67 (42.7)	10 (6.3)				227 (72.3)	87 (27.7)	0.55
**BRCA1+**	42	20 (47.6)	18 (42.9)	4 (9.5)		0.86	0.47	58 (69.0)	26 (31.0)	1
**BRCA2+**	57	29 (50.9)	24 (42.1)	4 (7.0)			0.78	82 (71.9)	32 (28.1)	1
**BRCA-**	58	31 (53.4)	25 (43.1)	2 (3.4)				87 (75.0)	29 (25.0)	0.47
**BRCA1/2+**	99	49 (49.5)	42 (42.5)	8 (8.0)			0.57	140 (70.7)	58 (29.3)	1

**Table 2:** Frequency of IVS3+24 ins16 (+) allele

		**Genotypic frequencies (%)**			***P *values**			**Allele frequencies (%)**		**HWE**
**Group**	**Sample number**	**- -**	**- +**	**+ +**	**All cases**	**BRCA2+**	**BRCA-**	**-**	**+**	***P *value**
**Controls**	112	79 (70.5)	32 (28.6)	1 (0.9)	0.65			190 (84.8)	34 (15.2)	0.46
**All cases**	157	102 (65.0)	53 (33.8)	2 (1.2)				257 (81.8)	57 (18.2)	0.11
**BRCA1+**	42	26 (61.9)	16 (38.1)	0		0.48	0.83	68 (81.0)	16 (19.0)	0.31
**BRCA2+**	57	38 (66.7)	17 (29.8)	2 (3.5)			0.46	93 (81.6)	21 (18.4)	1
**BRCA-**	58	38 (65.5)	20 (34.5)	0				96 (82.8)	20 (17.2)	0.19
**BRCA1/2+**	99	64 (64.7)	33 (33.3)	2 (2.0)			0.76	161 (81.3)	37 (18.7)	0.18

Haplotype analysis revealed that the TP53 polymorphisms are in linkage disequilibrium (D' = 0.80 – 0.93, for all test groups, p < 0.001), where the 72Arg and Ins16minus alleles occurred in strong disequilibrium. All possible double haplotypes were observed in the cases (Table 3), whereas there were no examples of Ins16-72Arg, Ins16-72Pro or Ins16-72Arg, Ins16minus-72Pro double haplotypes in the controls. However, there was only one example of each of these double haplotypes in the cases. Moreover, the distribution of the double haplotypes in the cases did not differ significantly from that of the controls. The paucity of some double haplotypes did not permit an analysis of all possible double haplotypes. However, notable is that the distribution of the Ins16minus-72Arg double haplotype in the cases (145 of 157, 92.4%) and controls (103 of 112, 91.7%) were similar (OR = 1.06 (95% CI 0.44 – 2.54), p = 1.

**Table 3:** Frequency of double haplotypes

		**Double haplotype frequencies (%)**									***P *value**		
**Group**	**Sample number**	**-G -G**	**-G +G**	**-G -C**	**-G +C**	**+G -C**	**+G +C**	**-C -C**	**-C +C**	**+C +C**	**All cases**	**BRCA2+**	**BRCA-**
**Controls**	112	55 (49.1)	2 (1.8)	21 (18.8)	25 (22.3)	0	0	2 (1.8)	6 (5.4)	1 (1.0)	0.98		
**All cases**	157	74 (47.1)	5 (3.2)	26 (16.6)	40 (25.5)	1 (0.6)	1 (0.6)	2 (1.2)	7 (4.5)	1 (0.6)			
**BRCA1+**	42	18 (42.9)	2 (4.8)	7 (16.7)	11 (26.2)	0	0	1 (2.4)	3 (7.1)	0		0.94	0.61
**BRCA2+**	57	26 (45.6)	2 (3.5)	12 (21.1)	11 (19.3)	1 (1.8)	1 (1.8)	0	3 (5.3)	1 (1.8)			0.34
**BRCA-**	58	30 (51.7)	1 (1.7)	7 (12.1)	18 (31.0)	0	0	1 (1.7)	1 (1.7)	0			
**BRCA1/2+**	99	44 (44.4)	4 (4.0)	19 (19.2)	22 (22.2)	1 (1.0)	1 (1.0)	1 (1.0)	6 (6.1)	1 (1.0)			0.61

### Genotype and Allele Frequencies of Hereditary and Familial Cases of Breast Cancer

The genotype frequencies of the TP53 polymorphisms in each of the three breast cancer cases groups based on BRCA mutation status were determined. Each of the BRCA1 and BRCA2 mutation-positive groups had the highest frequency of homozygous carriers of the 72Pro allele when compared with that of the mutation-negative group (Table 1). Homozygous carriers of the Ins16 allele were only observed in BRCA2 mutation-positive carriers (Table 2). However, neither of these differences nor the distribution of all genotypes were significantly different in all pair-wise comparisons of the three groups, or when the BRCA mutation-positive groups were combined in the analyses for each polymorphism.

The distribution of all possible double haplotypes in the cases is shown in Table 3. None of the breast cancer case groups had individuals with all possible double haploytpes. The highest frequency of carriers of the Ins16minus-72Arg haplotype, which is in strong disequilibrium, was within the mutation-negative group. The BRCA mutation-negative group had the highest frequency of carriers of the Ins16minus-72Arg haplotype (56 of 58, 96.6%). However, this observation was not significantly different from that observed in either of the BRCA1 mutation-positive (38 of 42, 90.5%) and BRCA2 mutation-positive (51 of 57, 89.5%) groups (p = 0.29).

### Age effects

**Figure 1:**
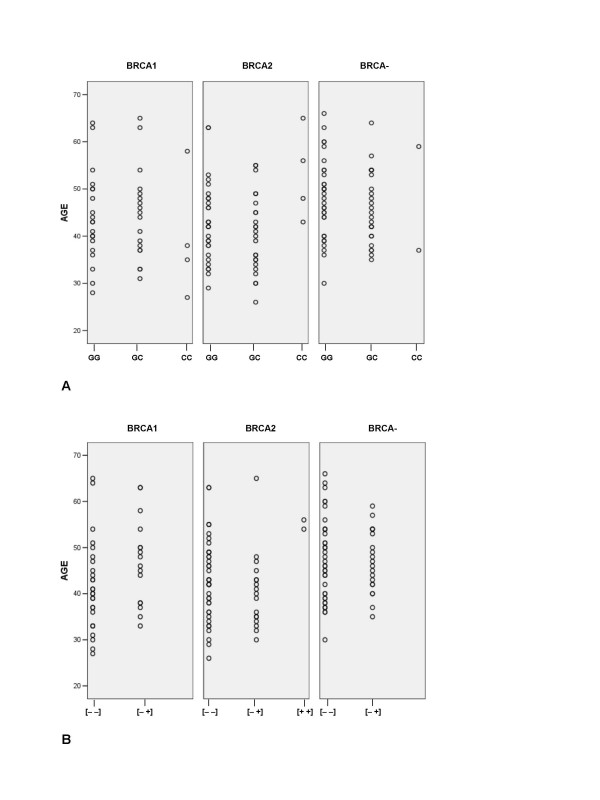
**Scatter plots of the age at diagnoses of breast cancer based on BRCA mutation status and genotypes of each TP53 polymorphism**. Panel A contains the age distribution of Arg72pro polymorphism based on BRCA mutation status for homozygous carriers of 72Arg (GG) and 72Pro (CC) genotypes or heterozygous carriers (GC). For the BRCA1 mutation-positive group, the G,G carriers (n = 20) had a mean age ± st. err.(standard error) = 43.9 ± 2.1; the GC carriers (n = 18) had mean age ± st. err. = 44.4 ± 2.3; and the CC carriers (n = 4) had a mean age ± st. err. = 39.5 ± 6.6. For the BRCA2 mutation-positive group, the GG carriers (n = 29) had a mean age ± st. err = 43 ± 1.6; the GC carriers (n = 24) had a mean age ± st. err. = 40.5 ± 1.7; and the CC carrier (n = 4) had a mean age ± st. err. = 53 ± 4.8. For the BRCA mutation-negative group, the GG carriers (n = 31) had a mean age ± st. err. = 48.1 ± 1.5; the GC carriers (n = 25) had a mean age ± st. err. = 46.0 ± 1.5; and the CC carriers (n = 2) had a mean age ± st. err. = 48 ± 11. Panel B contains the age distribution of Ins16 polymorphism based on BRCA mutation status for homozygous carriers Ins16minus [- -] and ins16 [+ +] genotypes or heterozygous carriers [- +]. For the BRCA1 mutation-positive group, the [- -] carriers (n = 26) had a mean age ± st. err. = 41.7 ± 1.9; the [- +] carriers (n = 16) had a mean age ± st. err. = 46.9 ± 2.3; and there were no homozygous [+ +] carriers. For the BRCA2 mutation-positive group, the [- -] carriers (n = 38) had a mean age ± st. err. = 43 ± 1.4; the [- +] carriers (n = 17) had a mean age ± st. err. = 40.5 ± 2.0; and the [+ +] carriers (n = 2) had a mean age ± st. err. = 55 ± 1. For the BRCA mutation-negative group, the [- -] carriers (n = 38) had a mean age ± st. err. = 47.3 ± 1.5; the [- +] carriers (n = 20) had a mean age ± st. err. = 46.9 ± 1.5; and there were no homozygous [+ +] carriers.

Overall, the BRCA mutation-negative cases had a significantly older mean age of diagnosis of breast cancer than each of the BRCA1 and BRCA2 mutation-positive groups (p = 0.02), as has been observed in previous studies of French Canadian breast cancer families [2]. The differences in age of diagnosis of breast cancer in the BRCA1 and BRCA2 mutation-positive groups did not permit the combined analysis of these groups versus mutation-negative group. We therefore investigated the ages of diagnoses of breast cancer within each group separately with respect to each TP53 polymorphism (Figure 1). Within the BRCA1 mutation-positive group, homozygous 72Pro carriers had the youngest ages of diagnoses of breast cancer than that of the homozygous 72Arg (p = 0.31) and heterozygous (p = 0.36) carriers, although these observations were not significant. Within the BRCA2 mutation-positive group, homozygous 72Pro carriers had a significantly older age of diagnosis of breast cancer compared to the mean age of diagnosis of either of the homozygous 72Arg (p = 0.041) and heterozygous (p = 0.018) carriers. Within the BRCA2 mutation-positive group, homozygous Ins16 carriers had a significantly older age of diagnosis of breast cancer compared to the mean age of diagnosis of either of the homozygous Ins16minus (p = 0.042) or heterozygous (p = 0.046) carriers.

**Figure 2:**
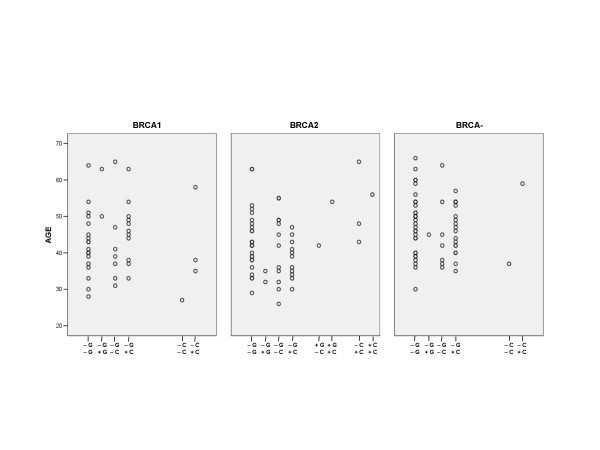
**Scatter plots of the ages at diagnoses of breast cancer based on BRCA mutation status and the double haplotypes observed for the TP53 polymorphisms**. Shown are the distributions of ages of diagnosis based on BRCA mutation status and TP53 polymorphism double haplotypes based on 72Arg [G], 72Pro [C], Ins16 [+] and Ins16minus [-] alleles. For the BRCA1 mutation-positive groups, the [-G; -G] carriers (n = 18) had a mean age ± st. err. = 42.6 ± 2.1; the [-G; +G] carriers (n = 2) had a mean age ± st. err. = 56.5 ± 6.5; the [-G; -C] carriers (n = 7) had a mean age ± st. err. = 41.9 ± 4.3; the [-G; +C] carriers (n = 11) mean age ± st. err. = 46.1 ± 2.5; the [-C; -C] carrier (n = 1) had a age = 27; and the [-C; +C] carriers (n = 3) had a mean age ± st. err. = 43.7 ± 7.2. For the BRCA2 mutation-positive carriers, the [-G; -G] carriers (n = 26) had a mean age ± st. err. = 43.5 ± 1.6; the [-G; +G] carriers (n = 2) had a mean age ± st. err. = 33.5 ± 1.5; the [-G; -C] carriers (n = 12) had a mean age ± st. err. = 41.8 ± 2.8; the [-G; +C] carriers (n = 11) had a mean age ± st. err. = 38.4 ± 1.6; the [+G; -C] carrier (n = 1) had a age = 42; the [+G; +C] carrier (n = 1) had a age = 54; the [-C; +C] carriers (n = 3) had a mean age ± st. err. = 52.0 ± 6.6; and the [+C; +C] carrier (n = 1) had a age = 56. For the BRCA mutation-negative group, the [-G; -G] carriers (n = 30) had a mean age ± st. err. = 48.2 ± 1.6; the [-G; +G] carrier (n = 1) had an age = 45; the [-G; -C] carriers (n = 7) had a mean age ± st. err. = 45.1 ± 3.9; the [-G; +C] carriers (n = 18) had a mean age ± st. err. = 46.4 ± 1.5; the [-C; -C] carrier (n = 1) had an age = 37; and the [-C; +C] carrier (n = 1) had an age = 59.

The low frequency of all double haplotypes did not permit an investigation of each possible combination with respect to age of diagnosis of breast cancer (Figure 2). However, we were able to analyze the combined data for carriers of the Ins16minus and 72Arg alleles as these alleles were in strong linkage disequilibrium. Within the BRCA2 mutation-positive group, carriers of the Ins16minus-72Arg haplotype had a significantly younger mean age of diagnosis of breast cancer (p = 0.009). Indeed the mean age of diagnosis of breast cancer at 41.7 years of the 51 Ins16minus-72Arg carriers was significantly younger than that of the six cases that did not carry this haplotype.

## Discussion

While there was an increased frequency of homozygous carriers of 72Pro alleles in BRCA1 and BRCA2 mutation carriers, and an increased frequency of homozygous carriers of Ins16 alleles in BRCA2 mutation carriers in familial breast cancer cases of French Canadian descent, the findings were not significant. The distribution of genotype frequencies was not significantly different in the controls or cancer cases in this study. Moreover, the distribution of genotypes and alleles of the unaffected controls and cases were comparable and not significantly different than that of European (or Caucasian) population as reported in the Single Nucleotide Polymorphism Database [49], a population that is historically or ancestrally linked with the French Canadians of Quebec [7]. These results suggest that there are no significant differences in the genotype frequencies in breast cancer cases regardless of the BRCA1 or BRCA2 mutation status. In independent reports of TP53 allele frequencies in cancer cases of BRCA1/2-mutation carriers, the relationships among carriers is not always apparent. In our study, in order to reduce bias due to the possibility of close familial relationships, we have purposely drawn our cases from independently ascertained families where (to our knowledge) the family members are not known to be directly related to each other [1-3]. However, our study size may be limited and further analysis of larger sample groups is warranted given the apparent increased frequency of homozygous carriers of rare alleles for both genotypes in BRCA1/2 mutation carriers when compared with mutation-negative cases.

Although there were differences in the ages of diagnosis of breast cancer in homozygous carriers of the 72Pro allele in BRCA1 and BRCA2 mutation positive groups, the mean age of diagnoses were not significantly different. BRCA2 mutation carriers homozygous for the 72Pro allele had an older mean age of diagnosis of breast cancer compared with breast cancer cases homozygous for the 72Arg allele and heterozygous carriers of this TP53 polymorphism, but overall the difference was not statistically significant. Although our studies were limited in size and are not significant, the younger mean age of diagnosis of breast cancer in BRCA1 mutation carriers homozygous for the 72Pro allele is consistent with independent reports suggesting that this allele may modify penetrance of BRCA1 carriers [47]. The independent report of the effect on age of diagnosis of breast cancer of the Ins16minus-72Pro haplotype in BRCA2 mutation carriers [45] was not observed in our study. However, we observed that among the BRCA2 mutation-positive cases, carriers of the Ins16minus-72Arg haplotype had a significantly younger mean age of diagnosis of breast cancer compared to the other individuals within this group. However, haplotype analysis suggested that the two polymorphic loci were in strong linkage disequilibrium in our samples. Although the extent of the linkage disequilibrium for the TP53 has not been properly investigated in this population, haplotype analysis of BRCA1 and BRCA2 loci of carriers of recurrent mutations have shown it could extend beyond at least one centriMorgan [1,3,6]. This observation was not surprising given the strong founder effects observed in the French Canadian population and the fact that the present day population are descendents of an estimated 8,500 settlers who colonized the present day St. Lawrence valley ("Nouvelle France") in recent history between 1608 and 1759 [7,8]. Hence, the young age of this founder population may also explain the relative paucity of individuals with the Ins16-72Arg haplotype in our sample groups.

## Conclusion

The analysis of TP53 alleles, 72Pro and Ins16, in French Canadians suggest that they do not significantly modify familial breast cancer risk. However our analyses may be affected by sample size and strong linkage disequilibrium observed in this population for the tested alleles. While additional breast cancer cases drawn from within each cancer families could have been included in our analyses as has been done in previous studies [25], this may further bias results due to the founder effects and linkage disequilibrium observed for the tested alleles in this population. However, the difference in homozygous allele frequencies of rare genotypes in this population warrants further investigation with a larger sample.

## Competing interests

The author(s) declare that they have no competing interest.

## Authors' contributions

LC carried out the molecular genetic and statistical analyses, participated in study design and drafted the manuscript. SLA participated in the molecular genetic analyses. CM, PG, A-M M-M and DP provided the clinical information of cancer families, BRCA1 and BRCA2 mutation status, and DNA samples from cancer families, and PNT conceived of the study, and participated in its design, statistical analyses and coordination, and drafted the manuscript. All authors read and approved the final manuscript.

## Pre-publication history

The pre-publication history for this paper can be accessed here:


